# Molecular Characteristics and Distribution of Virulence Genes among *Staphylococcus aureus* Complex Isolates Derived from Vascular Access Infections

**DOI:** 10.1155/2022/3196545

**Published:** 2022-10-21

**Authors:** Min Yi Wong, Yuan-Hsi Tseng, Tsung-Yu Huang, Chishih Chu, Bor-Shyh Lin, Yao-Kuang Huang

**Affiliations:** ^1^Division of Thoracic and Cardiovascular Surgery, Chiayi Chang Gung Memorial Hospital, Puzi, Chiayi 613, Taiwan; ^2^College of Photonics, National Yang Ming Chiao Tung University, Tainan 711, Taiwan; ^3^College of Medicine, Chang Gung University, Taoyuan 333, Taiwan; ^4^Division of Infectious Diseases, Department of Internal Medicine, Chiayi Chang Gung Memorial Hospital, Puzi, Chiayi 613, Taiwan; ^5^Department of Microbiology, Immunology, and Biopharmeceuticals, National Chiayi University, Chiayi 600, Taiwan

## Abstract

*Staphylococcus aureus* is a major human pathogen that produces various virulence factors which promote the binding of bacteria to tissues and medical devices such as vascular access devices, thereby developing a wide range of invasive infections. Vascular access serves as an entry site for *S. aureus* and elevates the risk of infection in the hemodialysis population. Nevertheless, the distribution of virulence genes in *Staphylococcus* spp. associated with vascular access infections (VAIs) has not been studied previously. In this study, we determined the relationship between the molecular characteristics and virulence profiles of *S. aureus* isolates obtained from VAIs. We collected isolates from patients with VAIs between August 2017 and December 2020 and further analyzed the molecular characteristics, antimicrobial resistance profiles, and virulence gene distribution in the isolates. Overall, 15 sequence types (STs), including a new ST (ST6892) and 19 *spa* types, were identified among the 56 isolates. Of the 53 *S. aureus* isolates, ST8, ST239, ST45, and ST59 were the predominant STs, whereas ST2250 was the only ST in 3 *S. argenteus* isolates. ST45-SCC*mec*IV-t026 (abbreviated as ST45-IV-t026), ST59-V-t437, and ST8-IV-t008 were the predominant clones that belonged to *agr* type I. All isolates harbored *clfB* and *eno*, whereas all *S. aureus* isolates harbored *clfA*. In addition, 10 Panton-Valentine leucocidin-positive isolates belonged to ST8 and ST59, with ST8-IV-t008 and ST59-V-t437 being the predominant clones. In brief, the distribution of virulence genes associated with STs may assist in the spread of molecular types of *Staphylococcus* spp.

## 1. Introduction

*Staphylococcus aureus* of the *Staphylococcus* genus is a major opportunistic pathogen capable of causing a wide variety of infections in humans. Humans serve as a natural host for *S. aureus* where approximately 30–50% of healthy adults are hosts, out of which 10–20% of the population shows persistent colonization [1]. *S. aureus* does not usually cause infection in healthy skin; only a breach of the skin or mucosal barrier provides entry into the bloodstream and internal tissues leading to potentially serious infections, such as bacteremia.

Different surface proteins of the microbial surface component recognizing adhesive matrix molecule (MSCRAMM) family mediate the adherence of bacteria to the host tissues, which is essential for the initial stage of infection [2]. MSCRAMMs are cell-wall anchored proteins that include fibronectin-binding proteins (FnbA and FnbB), fibrinogen-binding proteins (clumping factors ClfA and ClfB, and Fib), bone sialoprotein-binding protein (Bbp), laminin-binding protein (Eno), and collagen-binding proteins (Cna), which are covalently attached to peptidoglycan by sortase enzymes [3, 4]. *S. aureus* produces several exoproteins, including exfoliative toxins (ETA and ETB), leukocidin, and staphylococcal enterotoxins (SEs). The pyrogenic exotoxins, including SEs and toxic shock syndrome toxin-1 (TSST-1), are superantigens that can trigger T-cell activation to release cytokines [5]. Exoprotein expression is primarily regulated by the accessory gene regulatory (*agr*) quorum sensing system, which is environment-dependent and population density-dependent [6, 7]. The *S. aureus* exotoxin Panton-Valentine leukocidin (PVL) is a potent bicomponent pore-forming cytotoxin composed of LukS-PV and LukF-PV [8] and is mainly associated with tissue necrosis and leukocyte lysis [9].

A functional vascular access is necessary for efficient hemodialysis therapy because it directly affects the quality of life of hemodialysis patients. Both temporary and tunneled cuffed catheters, arteriovenous fistulas (AVFs), and arteriovenous grafts (AVGs) represent the commonly used vascular accesses [10]. However, the use of vascular access elevates the risk of contracting infections in hemodialysis patients, especially *S. aureus* infections, leading to access failure, infectious complications, and mortality [11].

We have previously studied the molecular characteristics of *S. aureus* associated with vascular access infections (VAIs) but not the distribution of virulence genes [12]. Therefore, this study aimed to determine the molecular characteristics of *S. aureus* isolates obtained from VAIs, evaluate the prevalence of virulence factors of *S. aureus* isolates, and clarify the correlation between VAI types, virulence genes, and genetic background.

## 2. Materials and Methods

### 2.1. Ethical Approval

The authors performed this study under the approval of the Institutional Review Board (IRB) of Chang Gung Memorial Hospital (IRB201508482B and IRB201901354B0). The authors collected the clinical information of patients by obtaining their written consent and conducted the study in accordance with approved guidelines.

### 2.2. Study Population and Sample Collection

The authors conducted the study at a tertiary teaching hospital in Chiayi, Taiwan (Chiayi Chang Gung Memorial Hospital), between August 2017 and December 2020. The authors collected 56 bacterial isolates from 43 hemodialysis patients with removal of infected tunneled‐cuffed catheters (TCCs), AVGs, and AVFs. The study procedure was explained to each patient individually before performing the procedures.

### 2.3. Bacterial Isolates Collection and Antimicrobial Susceptibility Testing

The bacterial isolates were derived from contaminated Hickman catheter tip, wound, pus, abscess, and blood and were cultured under laboratory standards. The samples were routinely cultured on blood agar at 37°C overnight. Strain identification was performed using standard biochemical (phenotypic) procedures. The isolates collected after 2019 were identified using matrix-assisted laser desorption/ionization time-of-flight (MALDI-TOF). The authors determined the antimicrobial susceptibility of *Staphylococcus* isolates via the disk diffusion method and interpreted the results in accordance with the standards of the Clinical and Laboratory Standards Institute [13].

### 2.4. Molecular Characterization

#### 2.4.1. Screening for MRSA and Staphylococcal Chromosomal Cassette *mec* (SCC*mec*) Type

The genomic DNA extraction was performed as previously described protocol [14]. To identify methicillin-resistant *S. aureus* (MRSA), the authors detected *mecA* using polymerase chain reaction (PCR) with previously described primer pairs [15]. The authors also identified SCC*mec* types I–V via a multiplex PCR assay using specific primers [16, 17]. The authors used sterilized water instead of template DNA as a negative control of PCR.

#### 2.4.2. Molecular Typing and Phylogenetic Analysis

To perform MLST for 56 isolates, the authors amplified the internal fragment of seven housekeeping genes using previously described protocol followed by sequencing [18]. When the *aroE* gene was unable to amplify, the authors used alternative primers described by Ruimy et al. [19]. Staphylococcal protein A (*spa*) gene typing was performed by amplifying the polymorphic *X* region using previously described protocol following sequencing [20, 21]. These amplified products sequencing in both directions were performed using the Sanger dideoxy DNA sequencing by Mission Biotech Co., Ltd (Taipei, Taiwan). The authors determined the MLST, *spa* types of each isolate, and an advanced grouping of MLST clonal complexes (CCs) using the BioNumerics software package ver. 7.6 (Applied Maths, Sint-Martens-Latem, Belgium) according to the MLST database [22] and Ridom Spa Server. The authors further performed the minimal spanning tree (MST) analyses based on MLST alleles with the categorical coefficient using BioNumerics software.

#### 2.4.3. *agr* Typing and Virulence Gene Characterization

For *agr* specificity groups I to IV identification, the authors amplified the hypervariable domain of the *agr* locus using specific primers as described previously [23]. The *pvl* gene was identified via PCR amplification, as reported previously [24]. The prevalence of MSCRAMM genes, including *clfA*, *clfB*, *fib*, *cna*, *fnbA*, *fnbB*, *bbp*, *ebps* (encoding elastin-binding protein), and *eno*, among the 56 isolates was evaluated via multiplex PCR as described previously [25]. The SE groups, including *sea*-*see*, *tst*, *etaA*, and *etaB*, were detected via PCR amplification using primer sets as described by Mehrotra et al. [26].

## 3. Results

### 3.1. Descriptive Characteristics of Patients with VAIs

In total, 43 patients with VAIs were enrolled in this study between August 2017 and December 2020. One female patient infected twice via two different vascular accesses in 2017 and 2019 was evaluated as two patients in subsequent analysis. The descriptive characteristics of the patients with VAIs are summarized in Table 1. Infections were more frequent in female patients than in male patients; infections associated with AVGs were predominant in female patients and TCC-associated infections were predominant in male patients. The median age among patients regardless of VAI type was approximately 66 years.

### 3.2. Molecular Characteristics of the SAC

In total, we collected 56 isolates from VAIs, including AVF (*n* = 3), AVG (*n* = 25), and TCCs (*n* = 28). Among the 56 isolates, 53 were *S. aureus,* and 3 were *S. argenteus*, a novel species closely related to *S. aureus* in the *S. aureus* complex (SAC), which was initially misidentified as *S. aureus* until we performed the following MLST typing.

#### 3.2.1. SCC*mec* Typing

Of the 56 SAC isolates, 30 isolates were *mecA*-positive MRSA, from which 1 isolate was oxacillin-susceptible (OS-MRSA), 23 were methicillin-susceptible *S. aureus* (MSSA), and 3 were methicillin-susceptible *S. argenteus* (MSSAg).

Overall, 30 MRSA isolates belonged to SCC*mec* types II to V and nontypeable (NT) SCC*mec* elements (Table 2). Among those MRSA isolates, SCC*mec* type IV was the most prevalent type, mainly originating from AVG infections, followed by SCC*mec* V, which mainly originated from TCC infections.

#### 3.2.2. MLST, *spa* Typing, *agr* Typing, and Phylogenetic Analysis

Among the 56 isolates, we identified 15 STs, including a new ST (ST6892). Specifically, ST8 and ST239, classified as CC8, along with ST45 and ST59, were the predominant STs. The only ST of *S. argenteus* isolates was ST2250; in contrast, the STs of *S. aureus* were more diverse. For instance, ST8, ST239, ST59, and ST45 were dominant in MRSA isolates, whereas ST15 and ST188 were dominant in MSSA isolates.

**Figure 1:**
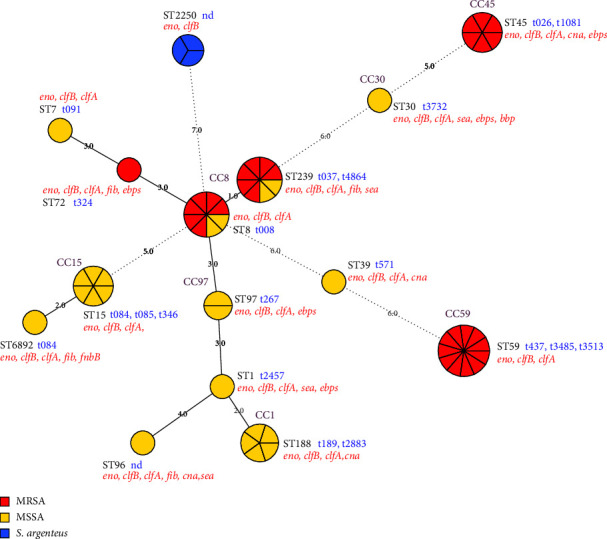
MLST minimum spanning tree (MST) analysis of the 56 *S. aureus* complex (SAC) isolates, including methicillin-resistant *S. aureus* (MRSA), methicillin-sensitive *S. aureus* (MSSA), and *S. argenteus*. Each color inside the circles represents the SAC (red, MRSA; yellow, MSSA; blue, *S. argenteus*). The *spa* type is indicated in blue font and the virulence factors are represented in red italic font. The lines connecting pairs of STs indicate their difference in alleles that one allele (thick lines), two alleles (thin lines), or three to seven alleles (dashed lines).

In total, we identified 19 *spa* types among the isolates, and t008, t437, and t026 were the predominant types. ST239-t4864 and ST8-t008 were identified in both MRSA and MSSA isolates. By comparing the correlation between the SCC*mec* type and the diverse MLST and *spa* types, we found that ST45-SCC*mec*IV-t026 (abbreviated as ST45-IV-t026), ST59-V-t437, and ST8-IV-t008 were the predominant clones in this study. The MLST MST of all strains based on their species is depicted in Figure 1.

According to *agr* typing analysis, 52 isolates were classified into *agr* specificity groups I to III, and 4 isolates, including 2 of 3 MSSAg isolates were nontypeable for the *agr* locus (Table 3). Of the 56 isolates, 75% (*n* = 42) included 28 MRSA, 13 MSSA, and 1 MSSAg isolates belonging to the predominant *agr* group I, followed by *agr* group II, which was only found in ST15 and ST6892 MSSA isolates. Furthermore, the prevalent ST59 and ST239 isolates all belonged to *agr* type I. This finding indicated that the *agr* types might be associated with the genetic background of the isolates. The distribution of the *agr* type among the AVG and TCC isolates was relatively uniform, indicating that the origin of VAI isolates showed no correlation with *agr* type distribution.

### 3.3. Distribution of Antimicrobial Resistance and Virulence Factors

#### 3.3.1. Antimicrobial Resistance Profiles of the Isolates

Overall, 95% of the 56 isolates were resistant to penicillin, including 100% of 28 TCC isolates and 92% of 25 AVG isolates. More than 50% of 56 isolates were resistant to erythromycin and oxacillin; the resistance rates to both antimicrobials were similar in AVG and TCCs isolates, with over 60% and 50% resistance to erythromycin and oxacillin, respectively. Approximately 38% of 56 isolates were resistant to clindamycin, mainly representing the AVG and TCC isolates. In contrast, the AVF isolates were only resistant to penicillin and oxacillin. This suggests that the antimicrobial resistance pattern of the VAI isolates was not directly correlated with the type of vascular access used.

Furthermore, among the MRSA isolates, ST59 and ST239 were multidrug-resistant (resistant to ≥3 antimicrobial classes) and included isolates resistant to clindamycin. In addition, 7 of 8 ST239 isolates showed resistance to trimethoprim-sulfamethoxazole (SXT); in contrast, ST8 isolates were only resistant to erythromycin, penicillin, and oxacillin.

#### 3.3.2. Virulence Factor Profiles

Of the 56 SAC isolates, all isolates harbored the *clfB* and *eno* genes, whereas *clfA* was found in all *S. aureus* but none of the *S. argenteus* isolates (Table 4). Neither *S. aureus* nor *S. argenteus* isolates harbored the *eta*, *etb*, *sed*, *see*, *tst*, and *fnbA* genes. The genes *ebps* and *cna*, and genes encoding enterotoxins including *sea*, *seb*, and *sec* were more prevalent in MRSA than in MSSA isolates; particularly, the *sec* gene was found in 17% (5/30) of the MRSA isolates, but not in any MSSA isolate. In contrast, more MSSA isolates carried *fib* and *fnbB* than those MRSA isolates. Approximately 50% of isolates harbored enterotoxin genes, in which *sea* was the predominant gene. All ST239 isolates harbored *sea*, whereas *sec* was only found in ST45 isolates. All ST239 MRSA isolates harbored the *cna* gene in contrast to the MSSA isolates. The relationship between genetic background and distribution of virulence factors is summarized in Table 5. ST8 and ST239 differed in only one allele based on the MST analysis (Figure 1); however, they showed considerable differences in phenotypic resistance and virulence gene profiles.

**Figure 2:**
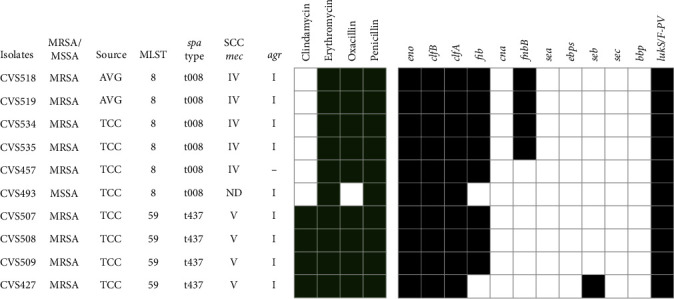
The molecular profile of PVL-positive strains (*n* = 10), including nine MRSA and one MSSA isolates. The phenotypic resistance is represented by a dark green square. The virulence genes, including enterotoxins and MSCRAMMs represent by a black square in the right panel.

We identified 10 PVL-producing isolates from VAIs, including 9 MRSA and 1 MSSA isolates (Figure 2). Those PVL-producing *S. aureus* belonged to ST8 and ST59. The PVL-producing MRSA isolates were SCC*mec* type IV and V, and *agr* type I and one NT *agr* type. The predominant *spa* types were t008 and t437 for ST8 and ST59, respectively. Therefore, the predominant clones of the PVL-producing *S. aureus* were ST8-IV-t008 and ST59-V-t437. As mentioned above, only the PVL-producing isolates belonging to ST59 were resistant to clindamycin; otherwise, they were only resistant to erythromycin, penicillin, and oxacillin. The predominant virulence genes in PVL-producing isolates were *clfA*, *clfB*, and *eno* as observed in other PVL-negative *S. aureus* isolates, whereas 80% (8/10) harbored the *fib* gene. In brief, the resistance and virulence profiles of PVL-producing *S. aureus* may depend on the ST of isolates; however, a bias might have developed owing to the small sample size in this study.

## 4. Discussion

Vascular access is required for efficient dialysis in patients; however, vascular access devices, regardless of the type, represent a common cause of infection in the dialysis population. Hemodialysis patients show an increased susceptibility to bacterial infections and have high infection or colonization rates with *S. aureus*. We previously found that *S. aureus* accounted for approximately 50% of isolates obtained from VAIs in our five-year single-institution study [14]. Despite the growing awareness of the clinical importance of *S. aureus* in VAIs, knowledge regarding its molecular architecture in the population is limited. In our previous study [12], we collected *S. aureus* isolates from wounds and abscesses around vascular access sites and infected vascular access sites in hemodialysis patients and further characterized *S. aureus* via MLST, *spa* typing, SCC*mec* typing, and biofilm formation ability. Due to the importance of virulence factors in the pathogenesis of *S. aureus*, we further analyzed the prevalence of virulence factors in *S. aureus* isolates obtained from VAIs in the present study and clarified the relationship between virulence factors, genetic background, and types of vascular access.

All *S. aureus* isolates possessed the *clfA* gene, similar to the studies in Hainan and Shanghai, China, where all collected *S. aureus* isolates harbor *clfA* [27, 28]. Another study on all MRSA isolates obtained from hemodialysis catheter-related bacteremia in Taiwan also harbor *clfA* [29], confirming that *clfA* may represent the most common virulence factor in *S. aureus*, not directly correlated to the regional distribution. In addition to the *clfA* gene, all *S. aureus* isolates in our study harbored the *clfB* and *eno* genes. Nemati et al. [30] reported that all poultry-derived *S. aureus* isolates possess the *clfA*, *clfB,* and *eno* genes, and Campoccia et al. [31] reported that all *S. aureus* isolates causing orthopedic implant infections harbor the *eno* gene, suggesting that these genes enable *S. aureus* to colonize hosts of various species. Besides, Contreras et al. [32] reported that MRSA isolates from catheter-related infections of ambulatory Mexican hemodialysis patients in their catheter and anterior nares predominantly harbor *clfA* (100%), *ebps* (96.9%), *clfB* (93.7%), and *cna* (93.7%). Compared to *S. aureus* from VAIs in this study, all isolates harbored *clfA* and *clfB*, but only 21% and 36% of *S. aureus* isolates harbored *ebps* and *cna*, respectively. The prevalence of virulence genes showed high similarity within the STs and was less relevant to infectious diseases or VAIs. For example, all ST45 isolates harbored *cna* and *ebps*, whereas all ST239 isolates harbored *fib* and *sea*. Notably, all ST239 MRSA isolates harbored *cna* in contrast to the ST239 MSSA isolates; however, *cna* was also found in MSSA isolates such as ST1, ST96, and ST188. In a previous study, Zamani et al. [33] reported that ST239 MRSA clinical isolates collected between 2018 and 2019 carried *cna*, while ST239 MSSA isolates from the same institution reported by Goudarzi et al. [34] contained no *cna* gene. However, Zamani et al. reported that all ST239 MRSA clinical isolates carried *clfA*, *clfB*, *fnbA*, and *fnbB*, which differed from our results where ST239 MRSA isolates harbored *eno*, *clfA*, *clfB*, and *fib*. The common virulence genes present within various lineages of *S. aureus* strains, suggesting their significant roles in staphylococcal pathogenicity, whereas others linked to specific molecular types may assist the spread of such molecular types.

PVL-producing *S. aureus* has been associated with skin and soft tissue infections, predominantly abscesses, and severe necrotizing pneumonia [9, 35]. So far, multiple studies have reported the presence of PVL in *S. aureus* circulating in hospital settings. A 2019 pediatric study in northern Taiwan [36] reported that the PVL genes were identified in 8.4% of isolates, 82% of which were characterized as ST59. Another study in Shanghai, China, [37] revealed that the PVL genes were identified in 30.8% (28/91) of all isolates from pediatric patients with SSTIs, with ST59 (50.0%, 14/28) as the predominant type. Besides, Chiu et al. [38] reported that 19.7% of clinical MSSA isolates in children were ST59 PVL-producing, 71.4% of which harbored the *seb* gene. In our study, we identified 17.86% of PVL-positive isolates (10/56) in the VAIs population, with ST8 (6/10) and ST59 (4/10) as the predominant types, wherein only one ST59 isolate carried the *seb* gene.

### 4.1. Study Limitation

We evaluated the distribution of virulence genes in VAI isolates in a single institution due to source limitations. However, the small sample size might have created a bias and limited the broad representation of the study. Nevertheless, the data still provide helpful information regarding the infection prevention and control and risk management of the hemodialysis population. Multicenter research can increase the sample size and provide accurate and conclusive results.

## 5. Conclusions

In summary, this epidemiological surveillance study elucidated the molecular characteristics, antimicrobial resistance profiles, and virulence factor distribution in SAC isolates predominantly present in VAIs. ST59, ST45, ST239, and ST8 were the predominant STs showing different patterns of antimicrobial resistance and virulence gene distribution. In brief, the distribution of antimicrobial resistance and virulence genes was correlated to molecular types, but not the types of infection, which may assist clinical treatment. A better understanding of the molecular characteristics and virulence gene distribution is necessary to improve clinical judgment and to establish an effective therapeutic strategy.

## Figures and Tables

**Table 1:** The descriptive characteristics of patients with *Staphylococcus aureus* complex (SAC) infections.

	VAI (*n* = 44)		
	AVF (*n* = 2)	AVG (*n* = 18)	TCCs (*n* = 24)
Sex of patients			
Male (n = 21)	0	3	14
Female (n = 27)^a^	2	15	10
Age (median ± SD)	66.85 ± 1.34	66.75 ± 9	66.3 ± 11.6

^a^One patient is counted as two episodes as one AVF in 2017 and one AVG in 2019.

**Table 2:** Molecular characterization of SAC isolates with SCC*mec* element.

	SCC*mec* (*n* = 30)				
	II (*n* = 3)	III (*n* = 3)	IV (*n* = 17)	V (*n* = 6)	NT (*n* = 1)
Origin					
AVF	0	0	2	0	0
AVG	3	1	10	0	0
TCC	0	2	5	6	1

**Table 3:** Frequency distribution of *agr* types in different SAC isolates.

	*agr* type			
	I (*n* = 42)	II (*n* = 7)	III (*n* = 3)	*agr*−(*n* = 4)
Species				
MRSA	28	0	0	2
MSSA	13	7	3	0
MSSAg	1	0	0	2
Origin				
AVF (*n* = 3)	2	0	0	1
AVG (*n* = 25)	19	3	2	1
TCCs (*n* = 28)	21	4	1	2
ST types	7 (1), 8 (7), 45 (5), 59 (11), 72 (1), 97 (2), 188 (5), 239 (8), 398 (1), 2250 (1)	15 (6), 6892 (1)	1 (1), 30 (1), 96 (1)	8 (1), 45 (1), 2250 (2)

**Table 4:** The distribution of virulence genes among molecular types of SAC isolates.

Virulence factors	ST type														
	1 (*n* = 1)	7 (*n* = 1)	8 (*n* = 8)	15 (*n* = 6)	30 (*n* = 1)	45 (*n* = 6)	59 (*n* = 11)	72 (*n* = 1)	96 (*n* = 1)	97 (*n* = 2)	188 (*n* = 5)	239 (*n* = 8)	398 (*n* = 1)	6892 (*n* = 1)	2250 (*n* = 3)
Leukocidin, enterotoxins															
*lukS-PV-lukF-PV*			6				4								
*sea*	1				1		2		1			8			
*seb*				3			7				1				
*sec*						5									

Adhesins															
*ebpS* (elastin-binding protein)	1				1	6		1		2					
*fib* (fibrinogen-binding protein)			7	5			5	1	1	1	4	8		1
*fnbB* (fibronectin-binding protein)			4	5						1		3		1	
*clfA*	1	1	8	6	1	6	11	1	1	2	5	8	1	1	
*clfB*	1	1	8	6	1	6	11	1	1	2	5	8	1	1	3
*eno*	1	1	8	6	1	6	11	1	1	2	5	8	1	1	3
*cna* (collagen-binding protein)						6			1		5	6	1		
*bbp* (bone sinloprotein binding protein)					1										

The following genes were not detected in any isolate: *sed, see, eta, etb, tst,* and *fnbA*.

**Table 5:** Correlation of ST type, *spa* type, SCC*mec*, *agr* type, and virulence factors with *S. aureus* isolates.

*S. aureus*	CC	ST	SCC*mec*	*spa*	*agr*	Virulence factors	No.
MRSA *n* = 30	8	8	II	t008	I	*eno, clfB, clfA, fib*	1
			IV			*eno, clfB, clfA, fib, fnbB, pvl*	4
					—	*eno, clfB, clfA, fib, pvl*	1
		72	IV	t324	I	*eno, clfB, clfA, fib, ebps*	1
		239	NT	t037	I	*eno, clfB, clfA, fib, cna, fnbB, sea*	1
			II	t4864		*eno, clfB, clfA, fib, cna, sea*	2
			III	t037		*eno, clfB, clfA, fib, cna, fnbB, sea*	2
						*eno, clfB, clfA, fib, cna, sea*	1
	45	45	IV	t026	I	*eno, clfB, clfA, cna, ebps, sec*	5
				t1081	—	*eno, clfB, clfA, cna, ebps*	1
		59	IV	t3485	I	*eno, clfB, clfA, fib, seb*	1
				t3513		*eno, clfB, clfA, seb*	2
				t437		*eno, clfB, clfA, sea, seb*	1
						*eno, clfB, clfA, sea, seb*	1
						*eno, clfB, clfA*	1
			V			*eno, clfB, clfA, fib, pvl*	4
						*eno, clfB, clfA, fib, seb*	1

MSSA *n* = 23	CC1	1	—	t2457	III	*eno, clfB, clfA, sea, ebps*	1
		188		t189	I	*eno, clfB, clfA, fib, cna*	2
						*eno, clfB, clfA, cna, seb*	1
				t2883		*eno, clfB, clfA, fib, cna*	2
	CC15	15		t084	II	*eno, clfB, clfA, seb*	1
						*eno, clfB, clfA, fib, fnbB*	1
						*eno, clfB, clfA, fib, fnbB, seb*	1
				t085		*eno, clfB, clfA, fib, fnbB, seb*	1
						*eno, clfB, clfA, fib, fnbB*	1
				t346		*eno, clfB, clfA, fib, fnbB*	1
	CC30	30		t3732	III	*eno, clfB, clfA, sea, ebps, bbp*	1
	CC8	8		t008	I	*eno, clfB, clfA, fib*	1
						*eno, clfB, clfA, pvl*	1
		239		t4864		*eno, clfB, clfA, fib, sea*	2
	CC97	97		t267	I	*eno, clfB, clfA, fib, fnbB, ebps*	1
						*eno, clfB, clfA, ebps*	1
		7		t091	I	*eno, clfB, clfA*	1
		96		NT	III	*eno, clfB, clfA, fib, cna, sea*	1
		398		t571	I	*eno, clfB, clfA, cna*	1
		6892		t084	II	*eno, clfB, clfA, fib, fnbB*	1

MSSAg *n* = 3		2250	—	nd	I	*eno, clfB*	1
					—		2

## Data Availability

The data are obtained from the corresponding author upon satisfactory request.
